# Potential for a global dynamic of Influenza A (H1N1)

**DOI:** 10.1186/1471-2334-9-129

**Published:** 2009-08-12

**Authors:** Antoine Flahault, Elisabeta Vergu, Pierre-Yves Boëlle

**Affiliations:** 1 EHESP School of Public Health, Rennes, France; 2 EHESP School of Public Health, Paris, France; 3 UMR S 707, INSERM, Paris, France; 4 MIA UR341, INRA, Jouy-en-Josas, France; 5 UMR S 707, Université Pierre et Marie Curie-Paris 6, Paris, France

## Abstract

**Background:**

Geographical and temporal diffusion patterns of a human pandemic due to Swine Origin Influenza Virus (S-OIV) remain uncertain. The extent to which national and international pandemic preparedness plans and control strategies can slow or stop the process is not known. However, despite preparedness efforts, it appears that, particularly in the USA, Mexico, Canada and the UK, local chains of virus transmission can sustain autonomous dynamics which may lead to the next pandemic. Forecasts of influenza experts usually rely on information related to new circulating strains.

**Methods:**

We attempted to quantify the possible spread of the pandemic across a network of 52 major cities and to predict the effect of vaccination against the pandemic strain, if available. Predictions are based on simulations from a stochastic SEIR model. Parameters used in the simulations are set to values consistent with recent estimations from the outbreak in Mexico.

**Results:**

We show that a two-wave pandemic dynamic may be observed in Southern hemisphere because of seasonal constraints for a maximum value of the basic reproductive number (R_0, max_) within a city equal to 1.5 and a mean generation interval (GI) of 2 days. In this case and in the absence of vaccination, attack rates may reach 46% when considering a completely susceptible population. More severe scenarios characterized by higher values of R_0, max _(2.2) and GI (3.1) yield an attack rate of 77%. By extrapolation, we find that mass vaccination in all countries (i.e. up to 50% of the population) implemented 6 months after the start of the pandemic may reduce the cumulative number of cases by 91% in the case of the low transmissible strain (R_0, max _= 1.5). This relative reduction is only 44% for R_0, max _= 2.2 since most of the cases occur in the first 6 months and so before the vaccination campaign.

**Conclusion:**

Although uncertainties remain about the epidemiological and clinical characteristics of the new influenza strain, this study provides the first analysis of the potential spread of the pandemic and first assessment of the impact of different immunization strategies.

## Background

Within 15 days of the WHO's raising the pandemic threat level to 6, more countries are affected by the new Swine Origin Influenza Virus (S-OIV) further raising concerns that S-OIV may be the next pandemic influenza strain. Active autonomous chains of transmission have been reported in several countries, such as Mexico, the USA, Canada, Spain and the UK. Most information about the virus and disease so far suggests a regular influenza process with many characteristics similar to those documented in past influenza pandemics [1]. Estimates of reproductive rates, higher than for seasonal influenza, are consistent with past pandemics [2,3]. Even if the possibility of a 1918-like scenario seems unlikely with the current circulating virus, the severity of the disease remains uncertain. Importantly, the underlying pathologies and causes of death in patients with S-OIV remain poorly documented, making the future burden of any pandemic uncertain. A major public health question as the disease continues to spread is identifying the likely course of the pandemic as well as possible control measures and their likely impact. Mathematical modelling has proven effective in retrospectively predicting the global circulation of the 1968–69 influenza pandemic, starting from Hong Kong and using coupled local epidemic processes [4,5]. Here, we aim at predicting the pattern of global spread of the potential S-OIV pandemic flu and at estimating the effect of vaccination campaigns under different scenarios.

## Methods

The model implements a metapopulation approach [4-6] where coupling between cities is through transportation (data on daily passengers flows from [7]). It simulates the spread of a pandemic through a worldwide network of 52 major cities. In each city, the progression of the disease is tracked by defining four disease states (Susceptible, Exposed, Infectious, Removed; *S, E, I, R*) and the transition rates between them. The exposed individuals (*E*) are not infectious and are assumed to travel whereas infectious individuals (*I*) do not travel. We adopt a stochastic framework in discrete time (with half-day time step), similar to [5], to capture effects of chance, especially at the source where the number of cases is still small. For each city *i *in the absence of any intervention, epidemic dynamics are described by the equations below (*t *tracks the time of the epidemic's spread at the population level whereas *τ *indicates the time since individual contamination):(1)

Coupling of local epidemic dynamics is described by population flows from city *i *to city *j *(*σ*_*ij*_). Transition probabilities between states are captured by distributions *γ(τ) *(from *E *to *I*) and *δ(τ) *(from *I *to *R*). *β*_*i*_(*t*) = Σ_*τ*_*βs*_*i*_(*t*)*I*_*i*_(*τ*,*t*)/*n*_*i *_is the probability that a susceptible individual becomes infected at *t+1 *and is proportional to the basic transmission rate (*β = R*_*0, max*_/*mean infectious duration*) modulated by the seasonality (*s*) and the proportion of infectious *(I/n)*. State variables in equation (1) are updated through random variables following Binomial distributions (*Bin*) which represent, in the order in which they appear in the equations: the number of new infections, the number of travelling latent subjects (in- and out-flows), the number of new infectious individuals coming from other cities, the number of new infectious individuals remaining in the city of origin, and the number of newly recovered individuals.

Parameter values were chosen according to qualitative knowledge or quantitative estimates. Consistent with early estimates of the basic reproductive number from data from the outbreak in Mexico [2,3], two plausible pandemic profiles were tested: in the first, a maximum (i.e. the value at the peak of transmissibility) basic reproductive number (R_0, max_) value of 1.5 was assumed [3] whereas in the second a higher R_0, max_, equal to 2.2, was chosen [2]. For the first case, the mean generation interval (GI) was assumed to equal 2 days (based on [3]) corresponding to mean exposed and infectious durations equal to 0.7 and 1.9 days respectively whereas in the second case, the GI was set to 3.1 (a value suggested in [2]) and the mean exposed and infectious durations were set equal to 1.45 and 2.9 days respectively. In both cases appropriate distributions of progression probabilities *γ(τ) *(with *τ*_*max *_equal to 1 and 2 for the first and second pandemic profiles respectively) and *δ(τ) *(with *τ*_*max *_equal to 4 and 7 respectively) were defined. As the average sojourn time in the exposed state for the first pandemic profile is less than a day, a time step of 0.5 days was adopted in order to correctly reproduce fast dynamic processes. The remaining parameters were identical for both pandemic profiles as detailed below. The observed seasonality in influenza transmission was incorporated using a step function: from October to March in the Northern hemisphere and the rest of the year in the Southern hemisphere the transmissibility (*s*) is maximum and equal to 1. The minimum value was set at 0.4. In the tropics, transmissibility was assumed constant over the year and equal to 0.7. Since no information is available on the existence of cross-immunity from past flu infections, the initial proportion of susceptible individuals was set to 100%. In agreement with current knowledge and data [3], the case fatality rate was assumed equal to 0.003. Deaths were counted but not subtracted from the number of infectious individuals since this does not appreciably impact on the dynamics of infectious individuals. Under-reporting was not addressed and no asymptomatic cases were considered. The pandemic originated in Mexico City where 20 cases were assumed to be present on 1 April.

Several scenarios for vaccination, introduced 6 months after the start of the pandemic, were tested for each of the two pandemic profiles. As little is known about the efficacy of a future vaccine, coverage and efficacy were combined into a unique intervention parameter through their product. Each vaccination scenario was defined by the duration of vaccination campaign and the number of cities where vaccination is implemented (all the cities or cities in developed countries only). Given that the objective is to immunize a predetermined proportion (*pv*) of susceptibles (with respect to the number of vaccine doses available at a given date) and that it seems reasonable to assume that only a maximum proportion (*α*, put equal to 0.01) of current susceptibles can be vaccinated daily, we fixed the duration (*d*) of the vaccination campaign at the value satisfying the equation *pv = 1-(1-α)*^*d*^.

First, we assumed that the vaccine was available in developed countries only and that the vaccination campaign lasted 15 days (corresponding to a proportion completely immunized in developed countries of 14%). In the second and the third scenarios, vaccination was implemented in all cities of the network over 35 and 70 days respectively (corresponding to global immunization rates of 30% and 50% respectively). Results are expressed as means calculated over 500 runs for each scenario.

## Results and discussion

**Figure 1:**
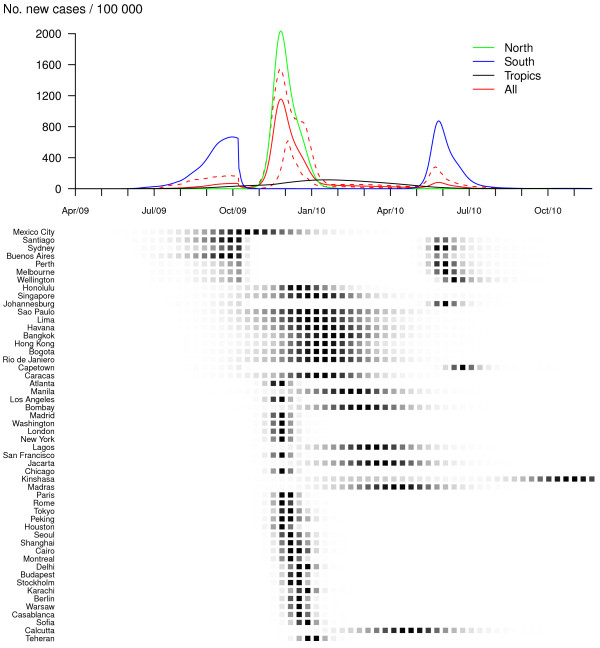
**Baseline scenario, no vaccination (R_0, max _= 1.5 and GI = 2)**. Dynamics of the pandemic starting from Mexico City, in late March 2009, in the absence of preventive and control measures. The upper panel represents average daily incidences for Northern (green), Southern (blue), tropical (black) and all (red) cities. Plain lines correspond to means and dashed lines (for the global curve only) to .05 and .95 pointwise quantiles calculated on 500 simulation runs. The lower panel illustrates the spread of the virus through the 52 cities of the network; the predicted probability of influenza activity is represented for each city (from 0 (white) to 1 (black)).

**Figure 2:**
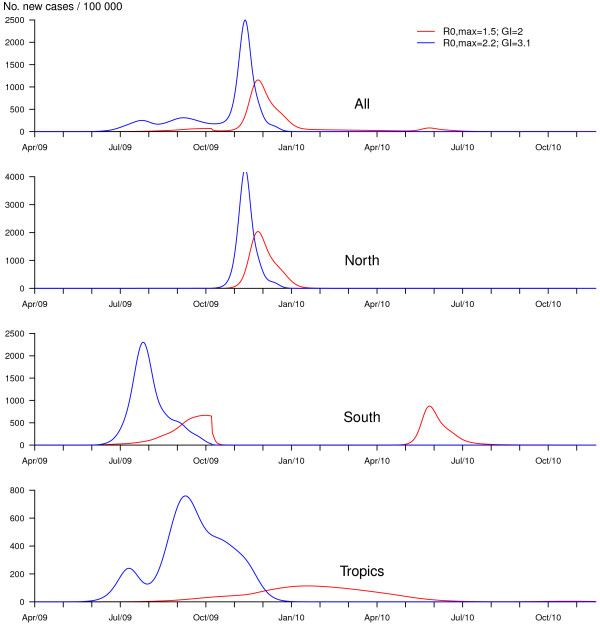
**Comparison between baseline scenarios (R_0, max _= 1.5 and GI = 2 versus R_0, max _= 2.2 and GI = 3.1), no vaccination**. Dynamics of the pandemic starting from Mexico City, in late March 2009, in the absence of preventive and control measures for two pandemic profiles (R_0, max _= 1.5 and GI = 2 (red); versus R_0, max _= 2.2 and GI = 3.1 (blue)). Graphs represent average daily incidences calculated on 500 simulation runs: for all cities of the network (upper panel) and specifically for cities in each zone (North, South and Tropics; lower panels).

According to simulations from our model including a seasonal forcing in flu transmission, for R_0, max _= 1.5 without any preventive or control measures, the pandemic would exhibit two waves (one in 2009 with a first Southern sub-wave and a second Northern sub-wave and the other in 2010), mainly owing to two successive epidemic events in Southern cities. In addition, the pattern would be different with respect to the zone considered (Figure 1). In contrast to the South, one massive epidemic would occur in the Northern hemisphere during the winter following the first Southern peak. In tropical cities activity would be moderate, spanning a much longer time period. The two-waves spread has indeed been described among the "signature features of influenza pandemics" [1]. In the case of a more transmissible viral strain (R_0, max _= 2.2), both the first Southern wave and the following Northern wave would be tremendous in size, affecting the vast majority of susceptible individuals of these zones (86%, Table 1 and Figure 2). In this case, the same virus would not spread the following year as a result of depletion of susceptibles.

**Table 1:** Forecasted total attack and mortality rates for two pandemic profiles (R_0, max _= 1.5 and GI = 2 versus R_0, max _= 2.2 and GI = 3.1)

Scenario	Total attack rate (%)		Total attack rate North (%)		Total attack rate South (%)		Total attack rate Tropics (%)		Mortality rate (%)	
	R_0, max _= 1.5 GI = 2	R_0, max _= 2.2 GI = 3.1	R_0, max _= 1.5 GI = 2	R_0, max _= 2.2 GI = 3.1	R_0, max _= 1.5 GI = 2	R_0, max _= 2.2 GI = 3.1	R_0, max _= 1.5 GI = 2	R_0, max _= 2.2 GI = 3.1	R_0, max _= 1.5 GI = 2	R_0, max _= 2.2 GI = 3.1
No vaccination	46	77	62	86	58	86	19	62	0.14	0.23
Vaccination in developed countries (14%)	37	71	47	74	55	86	17	62	0.11	0.21
Vaccination in all countries (30%)	9	51	11	45	32	86	2	52	0.03	0.15
Vaccination in all countries (50%)	4	43	1	31	32	86	2	52	0.01	0.13

Means of attack and mortality rates in the absence of any intervention and under different vaccination scenarios are calculated on 500 simulation runs for each scenario.

Owing to the scale used in Figure 1, influenza transmission during the first three months after pandemic onset is not visible on these graphs. However, simulated dynamics show early influenza activity with a daily worldwide incidence lower than 1/100000, which is consistent with the present situation (52160 cases of influenza A(H1N1) in 99 countries on 22 June 2009).

In the case of a moderately transmissible virus with R_0, max _= 1.5, 46% of the population would be infected worldwide by the end of 2010, mostly in Northern and Southern zones (Table 1), if no preventive or control measure were implemented. This proportion would increase to 77% in the case of a higher R_0, max _= 2.2. Although these attack rates may be over-estimated because of the assumption of an entirely susceptible and completely mixing population, the predicted values are not unrealistic compared with past pandemics. However, it is difficult to provide a more accurate prediction since no information is available on the existence of cross-immunity from past flu infections.

**Figure 3:**
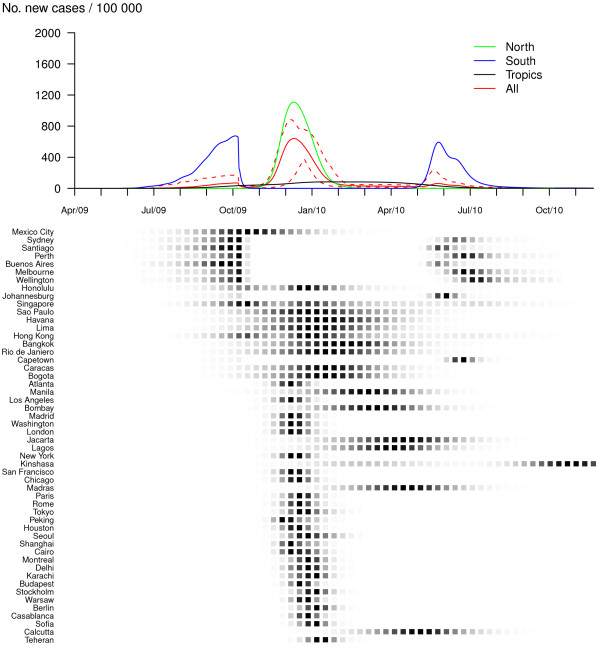
**Vaccination in developed countries only, 14% of population are immunized (R_0, max _= 1.5 and GI = 2)**. Dynamics of the pandemic starting from Mexico City, in late March 2009, with vaccine available only in developed countries 6 months after pandemic onset. Fourteen percent of the population in developed countries are vaccinated at a daily rate of 1%. The upper panel represents average daily incidences for Northern (green), Southern (blue), tropical (black) and all (red) cities. Plain lines correspond to means and dashed lines (for the global curve only) to .05 and .95 pointwise quantiles calculated on 500 simulation runs. The lower panel illustrates the spread of the virus through the 52 cities of the network; the predicted probability of influenza activity is represented for each city (from 0 (white) to 1 (black)).

**Figure 4:**
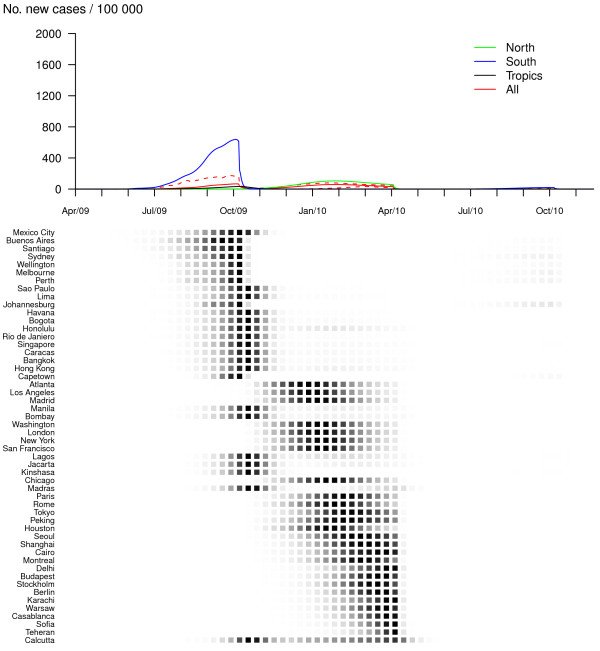
**Vaccination in all countries, 30% of population are immunized (R_0, max _= 1.5 and GI = 2)**. Dynamics of the pandemic starting from Mexico City, in late March 2009, with vaccine available in all countries 6 months after the pandemic onset. Thirty percent of worldwide population are vaccinated at a daily rate of 1%. The upper panel represents average daily incidences for Northern (green), Southern (blue), tropical (black) and all (red) cities. Plain lines correspond to means and dashed lines (for the global curve only) to .05 and .95 pointwise quantiles calculated on 500 simulation runs. The lower panel illustrates the spread of the virus through the 52 cities of the network; the predicted probability of influenza activity is represented for each city (from 0 (white) to 1 (black)).

**Figure 5:**
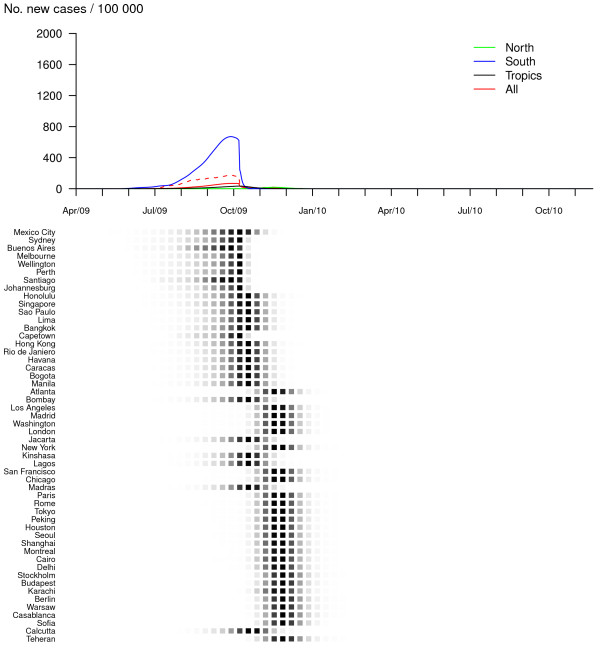
**Vaccination in all countries, 50% of population are immunized (R_0, max _= 1.5 and GI = 2)**. Dynamics of the pandemic starting from Mexico City, in late March 2009, with vaccine available in all countries 6 months after the pandemic onset. Fifty percent of worldwide population are vaccinated at a daily rate of 1%. The upper panel represents average daily incidences for Northern (green), Southern (blue), tropical (black) and all (red) cities. Plain lines correspond to means and dashed lines (for the global curve only) to .05 and .95 pointwise quantiles calculated on 500 simulation runs. The lower panel illustrates the spread of the virus through the 52 cities of the network; the predicted probability of influenza activity is represented for each city (from 0 (white) to 1 (black)).

The impact of vaccination differs according to the pandemic profiles and intervention scenarios. For the first pandemic profile (R_0, max _= 1.5 and GI = 2), making vaccine available in developed countries only and vaccinating 14% of the population does not change the global pattern of pandemic spread but reduces the global attack rate by 20% (Figure 3 and Table 1). The benefit, in terms of number of cases, is noteworthy in the Northern zone (where most developed countries are located) but is quite low in the Southern and Tropical regions (Table 1). In the third and fourth vaccination scenarios, where vaccination is implemented in all countries (30% and 50% of susceptibles are vaccinated respectively), the pandemic exhibits only a first wave and mainly in Southern cities (Figures 4 and 5). Mean global attack rates are significantly lower (9% and 4% respectively) and benefits are more homogeneously distributed among zones. However, the pandemic burden in the South is still important since the arrival of an effective vaccine 6 months after the start of the pandemic is unable to prevent the first wave in this region. Significant decreases in the global pandemic burden could be recorded for even smaller immunization proportions: if 20% (or 25%) of susceptibles are vaccinated, the mean global attack rate decreases to 21% (or 16%) (results not shown in Table 1).

**Figure 6:**
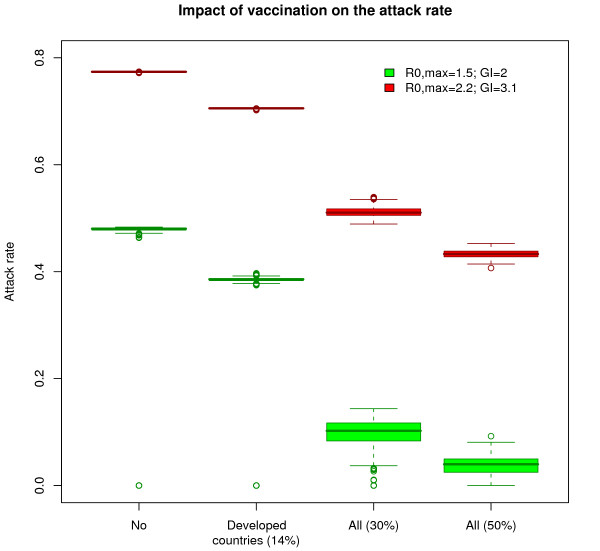
**Comparison of vaccination impact (R_0, max _= 1.5 and GI = 2 versus R_0, max _= 2.2 and GI = 3.1)**. Box-plots represent simulated distributions of attack rates over 500 simulation runs for two pandemic profiles and four vaccination scenarios (no vaccination; vaccination of 14% of population in developed countries only, vaccination of 30% of population worldwide and vaccination of 50% of population worldwide). The vaccine was considered available 6 months after pandemic onset.

The impact of vaccination is globally diminished in the case of a more transmissible influenza virus and a longer mean infectious duration (R_0, max _= 2.2 and GI = 3.1) (Table 1 and Figure 6). Even in the presence of a mass vaccination campaign (50% of population immunized), 43% of the population would still contract the infection. This is again explained by the fact that the large majority of cases (all the cases in the Southern cities) occur in the first 6 months, before the onset of vaccination.

In addition to intrinsic differences in the dynamics of the two pandemic scenarios (induced by different values of R_0, max _and of GI duration), the two-waves or one-wave patterns are partly due to seasonal forcing. As specified in the Methods, we considered that the transmissibility was 2.5 times greater during the influenza season in Northern and Southern zones (6 months in each hemisphere) than the rest of the year. In tropical regions the transmissibility was set to a constant throughout the year equal to 70% of the transmissibility during the influenza season in the North and South. The choice of a step function to represent variation in transmissibility and of the ratios between epidemic and non-epidemic seasons has a non-negligible impact on the simulated dynamic pattern. Further investigations are needed to evaluate their importance on the dynamics of the new circulating H1N1 strain.

All simulations are performed in the stochastic framework which allows capturing various effects of chance, especially at the source where the number of cases is still small. Results on final pandemic burdens, although based on stochastic runs, are quite stable and concentrated around the mean as illustrated in Figure 6.

As little information exists on the efficacy of any future vaccine, coverage and efficacy were summarized by a single parameter representing the proportion of the population effectively immunized. This approach is a rough approximation to reality and can be interpreted in several ways. For example, a 14% vaccine-induced immunity in the population may be the result of vaccinating 20% of population with a 70% effective vaccine or of vaccinating 70% of over 60 year-olds, assuming that the latter make up 20% of the population of developed countries. It will be possible to refine this approach as age-dependent initial natural immunity and transmissibility are further characterized. Beyond the specific case of vaccination, this kind of scenario could represent any preventive and control measure designed to protect susceptible individuals.

Finally, it is interesting to note that a scenario starting in Mexico City was already identified in previous work [8] exploring possible pandemic profiles. Here we refine our model used in [8] by adopting a stochastic framework and adapting this scenario to parameter estimates closer to those currently reported. Although the prediction concerning the Mexico City pandemic outlined in our previous study could have been altered by using a different classification method, it nevertheless remains that this scenario seemed to be a typical one. Our first prediction in [8] did not indicate a rule for identifying a pandemic-source location but it did provide some insight on how a pandemic would spread if starting in such a place. Ultimately, this finding was not surprising since Mexico City is well connected to the rest of the world and belongs to the tropical zone where viral strains circulate all year. These are two important characteristics for "successful" influenza spread.

## Conclusion

Although much remains to be done to characterize the new strain further, this study, based on models including estimates close to recently published data, shows that a multi-wave pandemic with a large attack rate is possible and may be curtailed using different immunization strategies.

## Competing interests

The authors declare that they have no competing interests.

## Authors' contributions

AF conceived the study. AF, EV and PYB developed the mathematical model. EV performed simulations. AF, EV and PYB analyzed the results. AF and EV drafted the manuscript. All authors read and approved the final manuscript.

## Pre-publication history

The pre-publication history for this paper can be accessed here:

http://www.biomedcentral.com/1471-2334/9/129/prepub
